# Supplementary material for: Transcriptome Analysis Reveals Vimentin-Induced Disruption of Cell–Cell Associations Augments Breast Cancer Cell Migration
Source: Cells. 2022 Dec 13;11(24):4035. doi: 10.3390/cells11244035 (PMC9776984; doi:10.3390/cells11244035)
Supplement: Supplementary file 1 [file cells-11-04035-s001.zip › DEGs and enrichment analysis.zip!/DEGs and enrichment analysis/WT_VIMvsVector GO enrichment/ALL/WT_VIMvsVector_all.dot.pdf]

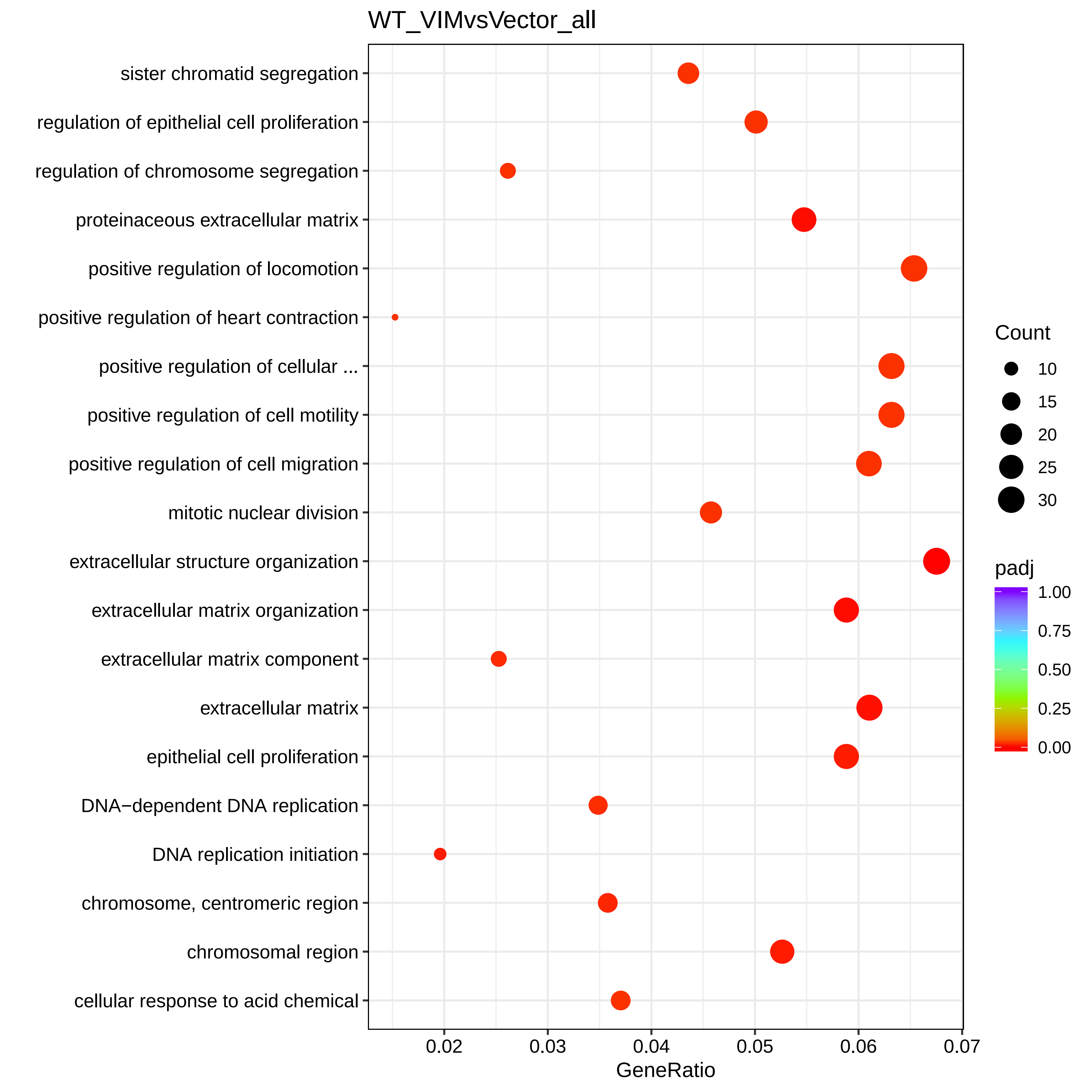
WT_VIMvsVector_all
sister chromatid segregation
regulation of epithelial cell proliferation
regulation of chromosome segregation
proteinaceous extracellular matrix
positive regulation of locomotion
positive regulation of heart contraction
positive regulation of cellular ...
positive regulation of cell motility
positive regulation of cell migration
mitotic nuclear division
extracellular structure organization
extracellular matrix organization
extracellular matrix component
extracellular matrix
epithelial cell proliferation
DNA−dependent DNA replication
DNA replication initiation
chromosome, centromeric region
chromosomal region
cellular response to acid chemical
0.02
0.03
0.04
0.05
0.06
0.07
GeneRatio
Count
10
15
20
25
30
padj
1.00
0.75
0.50
0.25
0.00
